# Dr. Stokes on Mayow's Discoveries

**Published:** 1800-04

**Authors:** Jonathan Stokes

**Affiliations:** Chesterfield


### To Dr. BRADLEY.

DEAR SIR,

DO you not think with me, that the admirers of Mayow, in their ardour to reſcue from a ſuppoſed oblivion the memory of an ingenious man, have attributed to him diſcoveries which belong belong indisputably to the chemists of the present day? I read him, nearly twenty years ago, in consequence of what was said of him by the translator of Scheele's Treatise on Air and Fire, who, I believe, was the late learned naturalist, Reinhold Forster; but, though I perused his work with much pleasure, I did not feel myself justified in saying more of him in my Thesis de Aëre Dephlogisticato, Edinb. 1782, than "quae in seculo praeterito de aëre dephlogisticato *auguratus est* iste ingeniosissimus Mayow, experimentis pulcherrimis comprobavit celeberrimus Priestley," p. 31. It is an abuse of language to call him the discoverer of an aërial fluid which he never saw, nor even attempted to obtain. Dr. Crane, in the Gent. Mag. for Jan. p. 48, goes farther back, and tells us, that Bathurst, so long ago as 1654, "knew what oxygen" gas "was," because he speaks of "*pabulum nitrosum*," et "*spiritus* aëris *nitrosus*." But, if such expressions are admitted as proofs of a knowledge of oxygen gas, I can go back to Sendivogius, who, according to Boerhaave, has said, that *the food of life* lies in the air;"* and Hohenheim, commonly known by the name of Paracelsus, who, in his Treatise de Morbis Metallicis, writes as follows, "Et veluti ventriculus cibum suum digerit, partim in corporis nutrimentum illum vertens, partim reliquias ex corpore exturbans: sic idem etiam de aëre judicandum est, *cujus pars una digeritur & consumitur, altera excernitur specie excrementi.*"†

The latest of the panegyrists of Mayow tells his reader, that he "will probably be surprised to find the following account of the formation of the acids in so old a writer; for it is exactly similar to the explanation which the new discoveries have given rise to." "The sulphuric acid, Mayow says, is formed *by the union of fire-air particles with the sulphur during combustion.*"‡ Mayow asserts no such thing; he supposes common sulphur to be a compound body, composed of pure sulphur and a certain salt of a fixed, or rather of a metallic nature. The particles of this salt, during the deflagration of the sulphur, he conjectures, are so altered by the mechanical action of the nitro-aërial particles, as to be changed from a solid to a fluid form, becoming the corrosive acid liquor known by the name of spirit of sulphur. "Suppono sulphur commune praeter particulas sulphureas puras putas, *salem quendam indolis fixae, seu potius metallicae*, particulis ejus sulphureis

---

* Boerh. Chem. by Shaw, i. 419. Boerh. Prael. ab Hallero, ii. 218, note. Obs. 141.

† Paracelf. Opera i. 707. fol. 1658.

‡ Observations on the claims of the moderns to some discoveries in chemistry and physiology, by G. D. Yeats, M. B. 1798, 8vo. p. 23.

ſulphureis ſtrictiſſime conjunctum continere; quae quidem pars ſalina nonnunquam in cryſtallos concreſcit, dum ſulphur a ſpiritu terebinthinae diſſolvitur. Porro annotandum eſt, flammam ſulphuris accenſi, uti etiam flammam quamcunque in eo conſiſtere, quod particulae materiae deflagrantis ſulphureae, et nitro-aëreae mutuo ſe motu velociſſimo exagitant. Jam vero cum particulae ſulphuris ſalinae minutiſſime diviſae, particulis ejus ſulphureis arctiſſime implicantur, fieri contigit, ut in ſulphuris deflagratione, (dum viz. particulae ejus ſulphureae et nitro-aëreae mutuo ſe motu igneo exagitant) *particulae ſulphuris ſalinae*, particulis ejus ſulphureis adhaerentes, *crebris particularum nitro-aërearum ictibus verberentur atterantur, comminuanturque*; ita ut *particulae eae ſalinae ſaepius attritae et contuſae, tandem inſtar gladiolorum exacuantur et inſuper adeo attenuentur, ut eaedem arigidis ſolidiſque in flexiles fluidaſque convertantur.* Particulae vero ſulphuris ſalinae, quae antea indolis fixae fuerant, poſtquam ita exacuantur et ad fluorem perducuntur, in liquorem acrem acidumque convertuntur; ſpiritumque ſulphuris vulgarem, uti veriſimile eſt, conſtituunt."*

Mayow regarded his ſpiritus nitro-aëreus, as one of his two ingredients of nitrous acid; but he did not conſider it as Lavoiſier did oxygen, as a component part of all the acids. He ſuppoſed it capable of combining both with acids and alkalies, and he imagined that its preſence increaſed their activity, and rendered them cauſtic. "Praeterea ſicut ſal acidum, ita etiam ſal fixum calcis vivae, (for he believed an acid and an alkali to exiſt in quick lime) ob particulas igneas in diuturna calcinatione ei infixas, ſumme mordax igneumque factum eſt. Etenim annotandum eſt, quod licet particulae nitro-aëreae igneaeque indolis ſalinae ſint, eae tamen neque ſali acido neque alcali contrariae ſunt; ſed e contra eorum alterutri combinatae *vires ejuſdem augent, igneaſque reddunt.*" p. 230. See alſo, p. 22. That he believed this combination not to be a chemical union, is evident from what follows. "Jam vero cum particulae nitro-aëreae ſali acido & fixo calcis, confertim infixae ſint, fieri contingit ut ſalia illa contraria particularum nitro-aërearum iis utriſque congruarum mediatione ab invicem detineantur, & veluti reconcilientur; ita ut ſe mutuo adoriri inque ſe invicem agere nequeant; dum vero ſalia iſta aqua diluta ſunt, *iſta particulas igneas ex parte ſaltem deponunt, & minus acria evadunt:* uti manifeſtum erit, ſi ſalia fixa igni violentiori commiſſa, poſtea in aqua ſolvantur; ita enim ſalia en, quae ab igne ſumme acria & cauſtica evaſerunt, acrimoniam deponent, & in

* Mayow Tractatus, 1674, p. 34.

priſtinum eſſe remigrabunt. Unde fit ut ſalia calcis contraria, poſtquam ea in aqua ſoluta ſunt, tum demum in ſe invicem agere, mutuoque effervefcere idonea ſint." p. 230. Theſe paſſages ſerve to explain what he means, when, ſpeaking of the acids, he ſays, "Quoad differentiam liquorum acidorum, eam a diverſitate ſalium, e quibus iidem conſtituuntur, procedere putandum eſt: uti etiam abeo, quod ſalia fixa nunc magis nunc vero minus a ſpiritu nitro-aëreo atterantur exacuenturque: & tamen inter ſalia acida quaecunque affinitas magna eſt & ſimilitudo; *inque iis omnibus particulae nitro-aëreae igneaeque veluti in ſubjecto idoneo hoſpitantur.*" p. 44. This paſſage, I apprehend, induced Dr. Yeats to ſuppoſe Mayow to have anticipated Lavoiſier in his theory of acidity, and to imagine that the former conſidered his ſpiritus nitro-aëreus to be a component part, not only of the nitrous but of all the acids. But if we attend to Mayow's account of the formation of the nitrous acid, we ſhall find him uſing a more preciſe language. "Ex iis quae dicta ſunt, haud difficile erit intellectu, quomodo ſpiritus acidus nitri in terra generatur. Etenim alibi oſtenſum eſt, terram fertilem nihil aliud eſſe quam ſulphur & ſal fixum, utraque immatura, arctiſſimo foedere invicem combinata; & utique terrae gleba atropurpurea colcothari haud multum ab ſimilis eſſe videtur; niſi quod in hoc ſulphur cum ſale metallico, in illa autem cum ſale fixo conjunctum ſit. Sicut ergo ſpiritus nitro-aëreus cum particulis ſulphuris vulgaris motu igneo effervefcens, item cum particulis ſalina ſulphureis colcotharis aeſtu magis remiſſo congreſſus, particulas eorum ſalino-metallicas citius aut tardius exacuit & ad fluorem perducit: ita ſpiritus idem nitro-aëreus pro penetrantiſſima ſua indole in terrae penetralia deſcendens, ibidem ſulphur terreſtre adoritur, cumque eodem motu obſcuro exaeſtuans, particulas ſalinas in ejus ſinu ſtrictius detentas atterit, attenuat, exacuitque, ita ut eaedem tandem flexiles liquidae *ſummeque acres* evadant. Particulae terrae ſalinae hoc modo ad fluorem evectae hoſpitium idoneum fiunt, in quo particulae nitro-aëreae recondantur detineanturque: *ab iis autem utriſque ſtrictim unitis* ſpiritum nitri, qualis diſtillatione elicitur, conſtitutum eſſe arbitror." p. 43. And, in his recapitulation, "Ex iis quae hactenus dicta ſunt, aliquatenus conſtare arbitror, e quibus ſal nitrum principiis componitur. Nempe videtur idem e ſale triplici conſtitutum eſſe; quorum alterum magis activum ab aere proſapiam ducit, idemque naturam aetheream igneamque obtinet; *ſal hoc Architectus ex materia terreſtri vehiculum ſalinum ſibi excudit, in quo veluti in ſubjecto idoneo hoſpitatur*; vehiculum illud ſalinum *una cum ſale igneo ſibi inſita* ſpiritum nitri conſtituit; qui mox ab ortu ſuo cum ſalibus terrae fixis, ad juſtam maturitatem perductis, congreditur; cumque que iisdem in nitrum vulgare coalescit." p. 46. And, again, " Et ita demum *Mercurius nitro-aëreus* pro furtiva sua indole, sulphuris hostis sui territoria clanculum ingressus, conjuge illud sua salina spoliavit, cui tandem *ipse tanquam sponsae idoneae maritatus, in illius amplexu pro infelici conjugii fato fixus & pene obrutus succumbit.*" p. 51.

Such is the theory of Mayow, in which he has been supposed by some to have anticipated Dr. Priestley in his discovery of oxygen gas, Mr. Cavendish in that of the component parts of nitrous acid, and the ever to be regretted Lavoisier in his theory of acids! Mayow's *spiritus nitro-aëreus* has been very generally considered as oxygen gas; and in my thesis I inserted it as a synonym of dephlogisticated air, not having then read his chapter De Luce, which contains the following passage: " Quoad medium cujus impulsu radii lucis transmittuntur, non est credendum illud ipsum aërum esse; siquidem lux etiam in vitro aëre vacuo, admodum intense propagari potest. Qua propter verisimile est, praeter particulas nitro-aëreas particulis aëreis infixas, particulas nitro-aëreas alias iisdem interspersas esse, earumque interstitia quaecunque adimplere. Id quod exinde colligimus, quoniam radii solares etiam in vitro ex quo aër exhauritur, ope vitri ustorii collecti, revera ignescunt. Etenim pulvis pyrius ab iisdem ibidem accendi, & etiam materia sulphurea eorum calore sublimari potest: calorem autem ignemque non nisi a particulis nitro-aëreis in motum concitis oriri, jam antea ostendere conatus sum. Ut videatur etiam *in loco aëre destituto particulas nitro-aëreas existere*; ignemque a radiis solaribus speculi ope coactis, ibidem conflatum, in eo consistere quod particulae nitro-aëreae in puncto illo in quo radii isti concurrunt, adeo impelluntur, ut eaedem in motum plane igneum concitantur." p. 199. And a little after, " Porro lucem a particularum nitro-aërearum impulsu propagari ex eo confirmari videtur, quod eadem per corpora ea facilius transmittitur, quae maxime obrigescunt, et particulis nitro-aëreis referta sunt; *cujusmodi sunt vitrum* idque genus alia, praesertim vero particulae aëreae, quarum obrigescentia a particulis nitro-aëreis ipsis confertim infixis provenit, prout antea ostendere conatus sum." p. 200. These passages appear to me decidedly to prove that the *spiritus nitro-aëreus* of Mayow is not the *oxygen gas* of modern chemistry; and that, if it be the prototype of any real existence, it must be that of *Calorique.*

But to return again to Dr. Yeats, who in relating Mayow's observations on the formation of acids, writes as follows: " Moreover the rust of iron, which possesses the nature of vitriol, appears to be produced by *the nitro-aërial particles attaching to the metallic sulphur of the iron.* " In this way he

 observes, obferves, ruft, or an imperfect vitriol, is produced, in the fame manner as if an acid had been thrown upon the iron. Has he not here anticipated the modern chemifts in their idea of an oxyd? p. 25." Mayow's words are, "Quin etiam Rubigo Ferri, quae naturam vitriolicam obtinet, particularum nitro-aërearum cum fulphure ferri metallico congredientium *actione* produci videtur: etenim *particulae falinae ferri modo praedicto ad fluorem perductae, particulas ejus metallicas corrodunt folvuntque*; ab iis vero utrifque" (that is, the faline and the metallic particles) "combinatis, rubigo five vitriolum quoddam imperfectum oritur, haud multo fecus ac fi ferrum liquore quovis acido oblitum fuiffet." p. 40. Hence it is evident that Mayow confidered the ruft of iron as a metallic falt, not a combination of oxygen and iron.

It has been generally underftood that Mayow conceived his fpiritus nitro-aëreus to be fecreted from the air, by the venous blood in the lungs. He appears indeed once to have thought fo; and we find him, in his Treatife de Refpiratione, expreffing himfelf as follows: "Et quidem verifimile eft, *particulas quafdam indolis nitro-falinae*, eafque valde fubtiles agiles fummeque fermentativas *ab aëre pulmonum minifterio fecerni*, inque cruoris maffam tranfmitti." p. 301. But in his Differtation de falnitro & fpiritu nitro-aëreo, publifhed fix years after, he appears to have changed his opinion, imagining the air to be abforbed by the lungs, and intimately mixed with the blood; and the nitro-aërial particles to be feparated from the reft by the fermenting particles of the blood, wearing down the aërial particles, and depriving them of the greater portion of their elafticity. "Super hoc aliquandiu fufpicatus fum, particulas nitro-aëreas elafticafque peculiari pulmonum contextura e particulis aëreis excuffas effe: verum cum ad rem diutius attenderam, potius vifum eft, *particulas aëreas in fanguinis maffam faceffere*; eafque ibidem particulis nitro-aëreis orbari, & proinde vim elafticam ex parte amittere." p. 136. "Aërem ab animalibus hauftum, modo fequenti vim elafticam putandum eft. Nempe imprimis fuppono maffam cruoris liquorem infigniter fermentefcentem effe, ut infra oftendetur. Quandoquidem ergo *particulae aëreae* pulmonum minifterio, *particulis ejus exaeftuantibus intime & quoad minima immifcentur*, fieri contigit, ut particulae aëreae haud fecus a particulis cruoris ac eaedem ab halitibus fermentefcentibus in vitro praedicto quoad vim elafticam imminuantur," (alluding to the experiment in which he extricated nitrous gas in a veffel containing common air, and in which Dr. Yeats has fo well explained the final depreffion of the water,*) 'Nimirum probabile eft, particulas fanguinis fermentefcentes,

* Yeats, p. 52.

scentes, *particulas aëreas iis interpositas atterere, spiritusque nitro-aëreas ex iisdem excutere*, atque eas demum particulis nitro-aëreis et elasticis privatas ad vitam sustinendam ineptas et insuper elatere suo ex parte destitutas fieri." How he gets rid of the remaining particles of air, I do not find. Lower, who, like Mayow and Thruston, adopts the language and theory of Bathurst, as mentioned above, deposits it on the solids, and finally carries it off by the pores of the body. "Si per quos pulmonum meatus *spiritus aëris nitrosus* in sanguinem transeat," says Lower, "eumque copiosius imbuat, a me quaeras, ostende et tu mihi quibus poralis alter *ille spiritus nitrosus qui in nive est*, per delicatulorum pocula transit et aestiva vina refrigerat, quod si vitrum aut metallum spiritui hinc non sint impervia, quanto facilius laxiora pulmonum vasa penetrabit? Denique si fuliginibus et seroso humori exitum non negemus; quidni per eosdem porulos vel similes, *nitroso hinc pabulo* introitum in sanguinem concedamus. Postquam autem *in habitu corporis et viscerum parenchymatis aër rursus a sanguine magna ex parte avolavit, atque per poros corporis transpiravit*, sanguinem venosum illo privatum obscuriorem et nigriorem illico apparere, rationi pariter consentaneum est." *p.* 5, 185, *ed.* 5, *Lugd. Bat.* 1708.

Hence I have been inclined to think, that Mayow gave two theories to the world; the first, in his Treatise on Respiration, which may be called the *saline theory* of Bathurst and Lower, if not of de le Boë Sylvius;* and in consequence of which Haller has placed him among those who held the doctrine of an aërial nitre;† and the latter, in his Tractatus Quinque, which may be stiled the *igneous theory*; and if Haller had studied it, he would have given him a place among those who held the doctrine of a vital principle in the air. What confirms me in the opinion, that in his first publication he merely adopted the nitrous theory of the day, is his frequent use of the phrase SAL *aëreum*, p. 301, 304, 305, SAL *nitro-aëreum*, p. 306, and "*particulae* NITRO-SALINÆ," p. 305;‡ whereas, in his chapter on the subject of respiration in his Tractatus Quinque, the terms

* Disp. Med viii. n. 77, in oper. p. 34. Praxis Med. lib. c. 21. p. 289.

† Elem. iii. 334, where the marks of reference to the notes *c* and *c** are transposed. Boerh. prael. ab Hallero, ii. 214.

‡ It is this theory which Connor makes the object of his criticism in his Diss. de Antris Lethif. 72, published in 1694, twenty years after the appearance of Mayow's Tractatus Quinque! If Connor had read this latter work, he did not understand it, for he says, "Nitrum aëreum *sal neutrum est & salsam de acido fixo particeps*." Nor does it appear from any thing Dr. Yeats has given us from Wolferstan, that he understood Mayow much better. Collins adopts his second theory in i. 26; but at p. 43 he gives a different hypothesis.

terms he uſes are, "SPIRITUS *nitro-aëreus*" and "*particulae* NITRO-AEREAE." If you ſhould have an opportunity of examining his *Tractatus duo, prior de reſpiratione, alter de rachitide*, publiſhed in 1668, and which I learn from the Phil. Tranſ. abr. iii. 80. was reviewed in the Tranſactions, No. 41, p. 833, I ſhall be obliged to you to inform me, in what reſpect it differs from the ſecond edition contained in the Tractatus Quinque, which were reviewed in No. 105, p. 104, as mentioned in the Phil. Tranſ. abr. iii. 225. From this laſt work, and from Hall. Elem. viii. 248, 249, I alſo find that the firſt edition of *Lower de corde, item de motu & colore ſanguinis*, appeared in 1669, the year after Mayow's Tractatus duo, ſo that it is poſſible that both works might be in the preſs at the ſame time. But at all events, Lower's poſition reſpecting the colour of arterial blood was ſo well eſtabliſhed by his own experiments, as not to ſtand in need of ſupport from any obſervations to be found in the firſt publication of Mayow, in which there are no experiments on that ſubject decidedly his own; for that at p. 299 of the Tractatus Quinque, is evidently Hooke's, which was publiſhed in the Phil. Tranſ. No. 28, and of which an account may be ſeen in Phil. Tranſ. abr. iii. 66, and in Med. Eſſays from the Ph. Tranſ. by Mihles, i. 18. and which Lower himſelf gives at p. 182, acknowledging himſelf indebted for it to Hooke.* I therefore join with Dr. Lubbock† in thinking Lower juſtifiable in not quoting Mayow; I muſt not however omit to mention, that Mayow quotes ſeveral of his cotemporaries, as Willis, Lower, Boyle, Des Cartes, Malpighi, Steno, Gliſſon, and Thruſton with due reſpect, the latter at p. 43 of the ſecond part of his Tractatus Quinque. But I do not obſerve that he has cited either

---

* This experiment was given in the firſt edition of the Treatiſe on Reſpiration, publiſhed in 1668, as appears from the citation of Etmuller, in *reſpirat. hum. negot. c.* 10. § 6; in *oper*. iii. 1879. This ingenious treatiſe of Etmuller, according to Haller, in elem. viii. 253, was firſt publiſhed in 4to. in 1676, as Dr. Lubbock, probably on the ſame authority, has already mentioned in your Journal, i. 418. That Etmuller underſtood the ſyſtem of Mayow, appears from his ingenious examination of it, which he thus introduces: "Conſideratius quidem de ſale quodam nitro-aëreo ad vitam ſuſtinendam neceſſario mentem ſuam proponit Mayowius de reſpirat. Licet non minus valdopere adhuc fluctuet. Nam. p. 301, uſum *particularum nitro-aërearum, quibus ex parte aërem conſtare* ſupponit, debitam dicit eſſe fermentationem in vaſis pulmonalibus & arteriis omnibus facere."—"Cum autem haec omnia gratis ſint alata, nec rationibus firmis munita; idcirco gratis eadem dimittere poſſemus, cum aſſenſum noſtrum in philoſophia naturali non niſi evidentes rationes ipſius materiae de qua agitur, crebris cautiſſimeque captis ac circumſpectiſſime applicatis experimentis fulcitae, nanciſci queant." *c.* 10, § 4, 5, *in Oper*. iii. 1877.

† In Med. & Phyſ. Journ. i. 419.

either de le Boë Sylvius, or Swammerdam, whose ingenious and experimental dissertation appeared in 1667. Dr. Yeats says, that "Swammerdam maintained that the more subtile part of the air was absorbed;"* but Haller enumerates him among those "qui *ipsum verum qualem ore adducimus aërem de atmosphaera in sanguinem venire* scripserunt,"† among whom also he ought to have enumerated Mayow. The following quotations seem to prove that Haller formed a just idea of the theory of Swammerdam. "Sanguis una cum aëre in pulmonibus dilatatis existens, facillime ab eo ventilari refrigerari at *ob subtilem hic iterum e sanguine egredientem*, (seu ab alio e corde in pulmones moto sanguine expulsam potius, et in aërem denuò ingredientem, imo et ipsum simul inflammantem,) *materiam* condensari, in se cogi, atque tandem *cum aëre misceri potest.* Quod praesertim eo facilius fieri posse concipimus, quia tunc temporis plurimi vapores calidi atque inutiles, una *cum expulsa subtili materia*, in aërem transeunt, atque illi admiscentur. In ea quae postmodum succedit expiratione, sanguis per pulmones motus, atque *propter subtilis materiae expulsionem, aërisque sive puri sive aliis infinitis modis alterati admistionem* refrigeratus condensatusque, ex arteriis pulmonalibus per venas, sinistrum versus cordis ventriculum porro propellitur. Imo, thorace se ulterius coarctante, pulmones exactius constringente, atque aerem quaquaversum premente et movente, et una cum fuliginibus seu vaporibus potius ex ipsis pulmonibus eum sanguinem expellente et propellente, *intimius is cum dicto aëre copulatur.*"‡ After giving an account of the farther progress of the blood towards the left ventricle, he adds: "Ita tandem, ob *admistum aërem* expulsosque vapores atque prae caeteris propter reiteratum *subtilioris materiae* affluxum, ad vitam conservandam, imo ad cor ipsum atque pulmones conservandos et nutriendos, ultimam ac postremam sanguinis adipiscitur perfectionem." What he means by this *materia subtilis*, one of the elements of Des Cartes, will best appear from the following passages. After adopting the acid and alkaline effervescence of the blood of his master De le Boë Sylvius, he says, "Propter illum itaque aestum seu motum, a sibi invicem oppositis non tantum et varie imo contrarie inter se motis agitatisve sanguinis particulis, sed et ob *subtilem materiam* praesertim, (*seu aëris partem tenuissimam per corporum poros facillime penetrantem, atque ex ipso aëre in* pectore ob hanc causam ut credimus semper praesente, a sanguine

* Obs. 103.

† Elem. iii. 320.

‡ De Respiratione, ed. 3, 1738, p. 38.

guine e corde continuo accedente *expulſam*, ſeu alias compedibus ſuis liberatam,) copia majori minorive, pro ſanguinis quantitate atque conditione, in ipſum continuo erumpentem & ingredientem, ſanguis vehementius, vel pacatius agitatur expanditurque." p. 37. How this ſubtile matter makes its way into the body, he farther explains in treating of the dilatation of the thorax: "Etenim *ſubtiliorem aliquam materiam* ob nimiam ſuam ſubtilitatem, pulmones appulſu ſuo minime extendere valentem, ſtatim *per carnes in pectus penetrare.*" p. 8.

Hence it appears, that Swammerdam, like Mayow, in what I call his ſecond or igneous theory of 1674, introduces the whole of the air into the maſs of blood; and as Mayow ſeparates his *ſpiritus nitro-aëreus*, Swammerdam lets looſe his *materia ſubtilis*. The admirers of Mayow will, perhaps, obſerve that Swammerdam makes his materia ſubtilis paſs alſo through the integuments of the body; but here too, I find, an analagous circumſtance in Mayow, where, treating of the fœtus in the egg, he ſays, "Itaque veriſimile eſt particulas nitro-aëreas *per incubentis fotum in ovum trajectas* ab humore ejus albugineo detineri;" and a little after, "Ita etiam *particulae eaedem nitro-aëreae* SUB CALORIS BLANDI SPECIE *ovi liquores ſubeuntes*, ad fermentationem vitalem motumque animalem in iiſdem inſtituendum, & proinde ad reſpirationis vicem praeſtandam, quodammodo conferre videntur." p. 325. Is not the *ſpiritus nitro-aëreus* another term for the *materia ſubtilis?*

I am, your's, &c.

